# Aqueous batteries: from laboratory to market

**DOI:** 10.1093/nsr/nwad235

**Published:** 2023-09-07

**Authors:** Xikun Zhang, Pengcheng Xing, Thomas L Madanu, Jing Li, Jie Shu, Bao-Lian Su

**Affiliations:** Laboratory of Inorganic Materials Chemistry (CMI), University of Namur, Belgium; Laboratory of Inorganic Materials Chemistry (CMI), University of Namur, Belgium; Laboratory of Inorganic Materials Chemistry (CMI), University of Namur, Belgium; Laboratory of Inorganic Materials Chemistry (CMI), University of Namur, Belgium; School of Materials Science and Chemical Engineering, Ningbo University, China; Laboratory of Inorganic Materials Chemistry (CMI), University of Namur, Belgium; State Key Laboratory of Advanced Technology for Materials Synthesis and Processing, Wuhan University of Technology, China

## Abstract

This perspective discusses the fundamental benefits and drawbacks of aqueous batteries and the challenges of the development of such battery technology from laboratory scale to industrial applications.

As estimated up to 2022, global installed capacity of lithium-ion batteries (LIBs) has increased dramatically from 148 to 580 GWh in the past five years. Their wide range of applications is mainly attributed to their high energy density, excellent cycle life, and low self-discharge rate [1]. However, with the continuous promotion of the applications in various fields, the limitations of LIBs have become apparent, such as high cost and safety issues. The search for a sustainable alternative to LIBs has gathered pace in order to meet the demand for highly efficient energy storage while simultaneously ensuring safety. Aqueous batteries have gradually entered the stage of energy storage systems due to their low cost and high safety [2]. Driven by the need for safer and more efficient energy storage, aqueous batteries attract significant research attention. However, their energy density and cycling performance are not currently satisfactory enough, impeding their industrial application.

Research interest in aqueous batteries, which is increasing year by year (Fig. 1A), is mainly focused on the optimization of electrode materials and electrolytes. Compared with non-aqueous batteries, aqueous batteries have inherent superiority in terms of safety, cost-effectiveness, high conductivity, and ease of manufacturing process (inset of Fig. 1A). The electrochemical reaction inside an aqueous battery is a complex multi-step process (Fig. 1B). Initially, a charge carrier in the electrolyte undergoes a solvation and ion migration process in order to reach the vicinity of the electrode. Subsequently, a desolvation process occurs on the surface of the electrode material before electrochemical reaction. Finally, the charge carrier adsorbs on the electrode material and then inserts into the structure. Therefore, the composition of electrolyte, the migration of charge carrier, the solvation/desolvation process, and the electrochemical reaction of charge carrier with electrode materials are all critical to the electrochemical performance of aqueous batteries.

**Figure 1.**
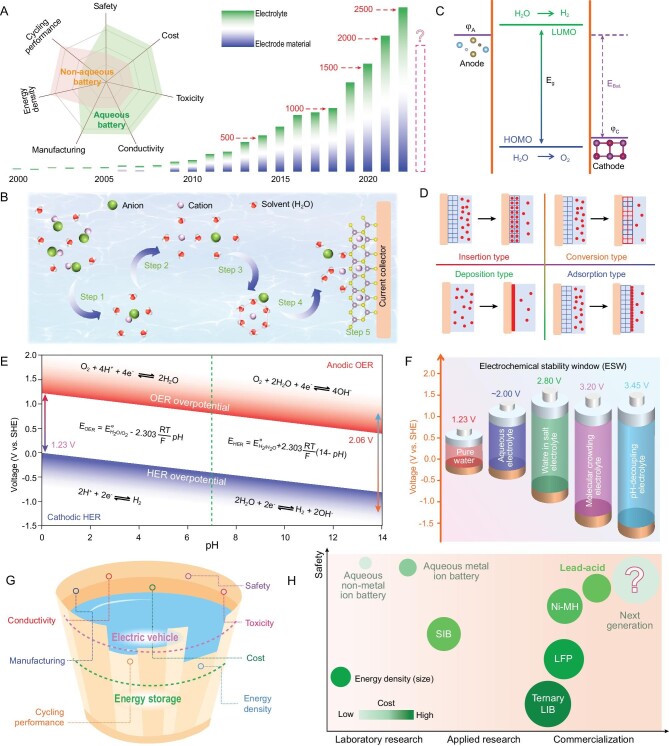
(A) Number of publications for aqueous batteries and the ratio between research on electrolytes and electrode materials. Data were collected from Web of Science in December 2022. Inset depicts a comparison of the superiority of aqueous and non-aqueous batteries. (B) Electrochemical reaction process in aqueous batteries. Step 1: salt dissolution; Step 2: solvation process; Step 3: ion migration; Step 4: desolvation process; Step 5: insertion process. (C) Schematic diagram of the relationship between the energy level of electrode and electrolyte. In aqueous batteries, φ_C_ should be higher than the HOMO of electrolyte and φ_A_ should be lower than the LUMO, failing which irreversible solvent decomposition reactions occur. (D) Four different types of electrochemical reactions in aqueous batteries. (E) Pourbaix diagram of water in aqueous electrolyte with different pH values and possible OER/HER processes. (F) The electrochemical stability window in different aqueous electrolytes. (G) The superiority and inadequacy of aqueous batteries for future large-scale energy storage systems and movable power battery of electric vehicles. (H) The comparison between aqueous batteries and commercial batteries regarding safety, energy density, cost, and technology.

The energy density of aqueous batteries is mainly restricted by the limited capacity and operating voltage. The properties of electrode materials and electrolytes, as well as their interactions, are therefore crucial to realize aqueous batteries with high energy density. In terms of electrode materials, their electrochemical reaction with charge carriers determines the capacity and operating voltage of the battery. Moreover, the properties of the electrolyte decide the electrochemical stability window (ESW) of the aqueous battery. Regulation of the electrolyte is thus also crucial to improve energy density. In addition, considering their practical application, some common problems faced by commercial batteries also need to be solved in aqueous batteries, such as the difference in battery design between the laboratory and the market, working in extreme operating environments, etc.

The voltage matching principle between the electrode material and electrolyte in aqueous batteries is depicted in Fig. 1C. Generally, the electrode potential of the cathode reaction (φ_C_) should be higher than the highest occupied molecular orbital (HOMO) of electrolyte and the electrode potential of the anode reaction (φ_A_) should be lower than the lowest unoccupied molecular orbital (LUMO) [3]. In non-aqueous batteries, a protective solid electrolyte interphase layer is always formed on the surface of the electrode material due to the decomposition of organic electrolyte. Different from non-aqueous batteries, the surface film in an aqueous battery is usually attributed to the deposition of by-products on the electrode surface, which are generated by the hydrolysis reaction of intermediates or final products in aqueous solution, and the electrolyte does not participate in the reaction. The as-formed by-products on the electrode surface will bring over-potentials for both cathode and anode, thus inducing charge-discharge voltage hysteresis and reducing the practical output voltage of an aqueous battery.

Due to different couples of charge carriers for electrode materials, there are different types of reaction in aqueous batteries, such as insertion, conversion, deposition, and adsorption reactions (Fig. 1D) [4]. Generally, conversion and deposition reactions tend to yield higher capacity but produce lower working voltage. It is known that higher capacity and working voltage represent higher energy density at the same conditions, which is beneficial in practical applications that require high energy density. However, the conversion reaction always leads to severe volume expansion or even pulverization of electrode material, which is detrimental to the cycling performance of the battery. On the contrary, insertion and adsorption reactions can produce higher working voltage and excellent cycling performance, despite the capacity being partially sacrificed. It is crucial for aqueous batteries to maintain an intricate balance between energy density and cycling performance in practical applications.

At present, a series of optimization measures have been proposed for electrode materials to enhance their energy density. For example, pre-intercalation chemistry methods can be applied to enlarge the diffusion path of charge carriers and create more active sites inside electrode materials, which could thus enhance the specific capacity for insertion-type hosts [5]. Surface engineering is also applied to fabricate electrode materials with sluggish hydrogen evolution reaction (HER) and oxygen evolution reaction (OER) kinetics for developing high-voltage aqueous batteries. Taking Mn_5_O_8_ as an example, bare Mn_5_O_8_ exhibits a stable potential window of 2.5 V, but surface hydroxylated Mn_5_O_8_ shows a higher operating potential range of 3.0 V in the same electrolyte [6]. Material modification engineering is therefore effective for improving the energy density of aqueous batteries.

The nature of the electrolyte is another crucial factor. In aqueous batteries, electrolyte is formed by dissolving inorganic salt in water. Taking water as a solvent, aqueous electrolyte not only has the advantages of low cost, non-flammability, and non-toxicity, but also offers higher ionic conductivity beneficial to ion migration, which is two orders of magnitude higher in the case of aqueous electrolyte compared to that of organic electrolyte [7]. Although water is an excellent solvent for electrolytes, its narrow splitting voltage window greatly limits the energy density of aqueous batteries. In pure water, the splitting voltage window is 1.23 V. Considering the OER and HER overpotential caused by salt concentration and pH, the voltage window of aqueous electrolyte can be slightly expanded to 2.06 V, which still does not meet the demand for high energy density [8]. Moreover, as shown in Fig. 1E, pH has a significant influence on OER and HER reactions [9]. In either acidic or basic conditions, OER and HER reactions in the electrolyte are unavoidable. Research on solutions to widen the ESW of aqueous batteries is currently in progress.

To widen the ESW of aqueous batteries, various solutions have been proposed (Fig. 1F). First, water-in-salt (WIS) electrolyte is used to broaden the ESW of aqueous batteries. The number of free water molecules can be reduced by using high concentration or ultra-high concentration electrolytes, thereby reducing the chemical activity of water molecules, destroying the hydrogen bonds of water molecules, and regulating the solvation structure of charge carriers. As a result, the ESW was successfully widened to 2.6 V [10]. Second, molecular crowding electrolyte further broadens ESW to 3.2 V. Different from WIS electrolyte, molecular crowding electrolyte forms a molecular crowding environment through the additives in the electrolyte thereby reducing reaction activity of water molecules [11]. The above approaches achieve the purpose of reducing the chemical activity of water molecules through weakening the relationship between water molecule and charge carrier, and successfully broaden the ESW. Last, the pH-decoupling electrolyte separates the acidic catholyte and basic anolyte by an ion exchange membrane and inhibits the chemical crossover between them, realizing the cathode working in acidic catholyte and the anode working in basic anolyte, respectively [12]. The ESW was effectively increased to 3.45 V. Although the current energy density of aqueous batteries cannot meet the required demand, next-generation aqueous batteries with high energy density and excellent cycling life could certainly be realized via effective regulation of electrode materials and electrolytes in the near future.

In addition to the rational design of electrode materials and electrolytes, the following aspects should also be considered in the practical applications of aqueous batteries. First, compared with laboratory studies, the usage amount of electrolyte in practical aqueous batteries will be greatly reduced. It is therefore crucial to maximize the electrochemical performance of electrode materials with limited electrolyte usage. Second, aqueous batteries should make optimal use of its advantages of high ionic conductivity to achieve fast energy storage (fast charging capability). At the same time, the possible increase in OER and HER cannot be ignored at high rates. Third, aqueous batteries must have the ability to work in extreme operating environments, such as high ambient temperatures and severe deformations. So far, through the regulation of electrolytes, aqueous batteries can operate in a wide temperature range from −90 to 80^o^C. Finally, to promote its application in flexible electronics, aqueous flexible batteries are expected to be developed in the future.

In short, the energy density and cycling performance of current aqueous batteries have met the demand of energy storage systems to a certain extent (Fig. 1G). There is still a gap to catch up the requirement of rechargeable batteries for electric vehicles. Nevertheless, an aqueous battery has the advantages of low cost, high ion conductivity, non-flammability, and non-toxicity. With the continuous development of electrode and electrolyte engineering, next generation aqueous batteries are expected to become the protagonist of commercial batteries (Fig. 1H).
